# Structure-activity relationship analysis of cytotoxic cyanoguanidines: selection of CHS 828 as candidate drug

**DOI:** 10.1186/1756-0500-2-114

**Published:** 2009-06-29

**Authors:** Henrik Lövborg, Robert Burman, Joachim Gullbo

**Affiliations:** 1 Division of Clinical Pharmacology, Faculty of Health Sciences, Department of Medicine and Care, Linköping University, SE-581 85 Linköping, Sweden; 2 Division of Pharmacognosy, Department of Medicinal Chemistry, Uppsala University, Biomedical Centre, Box 574, SE-751 23 Uppsala, Sweden; 3 Division of Clinical Pharmacology, Uppsala University Hospital, SE-751 85 Uppsala, Sweden

## Abstract

**Background:**

*N*-(6-(4-chlorophenoxy)hexyl)-*N*'-cyano-*N''*-4-pyridyl guanidine) (CHS 828) is the first candidate drug from a novel group of anti-tumour agents – the pyridyl cyanoguanidines, shown to be potent compounds interfering with cellular metabolism (inhibition of nicotinamide phosphoribosyl transferase) and NF-κB signalling. Substituted cyanoguanidines are also found in anti-hypertensive agents such as the potassium channel opener pinacidil (*N*-cyano-*N'*-(4-pyridyl)-*N''*-(1,2,2-trimethylpropyl)guanidine) and histamine-II receptor antagonists (e.g. cimetidine, *N*-cyano-*N'*-methyl-*N''*-[2-[[(5-methylimidazol-4-yl]methyl]thio]ethyl)guanidine). In animal studies, CHS 828 has shown very promising activity, and phase I and II studies resulted in further development of a with a water soluble prodrug.

**Findings:**

To study the structural requirements for cyanoguanidine cytotoxicity a set of 19 analogues were synthesized. The cytotoxic effects were then studied in ten cell lines selected for different origins and mechanisms of resistance, using the fluorometric microculture cytotoxicity assay (FMCA). The compounds showed varying cytotoxic activity even though the dose-response curves for some analogues were very shallow. Pinacidil and cimetidine were found to be non-toxic in all ten cell lines. Starting with cyanoguanidine as the crucial core it was shown that 4-pyridyl substitution was more efficient than was 3-pyridyl substitution. The 4-pyridyl cyanoguanidine moiety should be linked by an alkyl chain, optimally a hexyl, heptyl or octyl chain, to a bulky end group. The exact composition of this end group did not seem to be of crucial importance; when the end group was a mono-substituted phenyl ring it was shown that the preferred position was 4-substitution, followed by 3- and, finally, 2-substitution as the least active. Whether the substituent was a chloro, nitro or methoxy substituent seemed to be of minor importance. Finally, the activity patterns in the ten cell lines were compared. Substances with similar structures correlated well, whilst substances with large differences in molecular structure demonstrated lower correlation coefficients.

**Conclusion:**

According to this structure-activity relationship (SAR) study, CHS 828 meets the requirements for optimal cytotoxic activity for this class of compounds.

## Background

**Figure 1:**
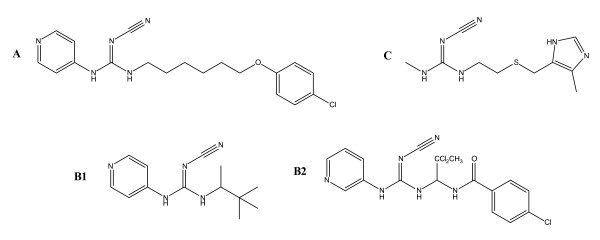
**Substituted cyanoguanidines with defined pharmacological effects**: **A **Cytotoxic CHS 828; **B **Potassium channel openers pinacidil (**B1**) and 12 g of compound as described in Perez-Medrano *et al *[2] (**B2**); and **C **Histamine-II receptor antagonist cimetidine.

The anti-tumoral activity of the pyridyl cyanoguanidines was first detected in a routine *in vivo *screening programme in a rat model with Yoshida ascites sarcoma cell tumours. The studied compounds were synthesized as analogues to the anti-hypertensive potassium channel opener pinacidil (Fig. 1). Replacement of the side chain by longer aryl-containing side chains caused a loss of activity in this respect, and a candidate drug, *N*-(6-(4-chlorophenoxy)hexyl)-*N'*-cyano-*N''*-4-pyridyl guanidine) (CHS 828), was selected after studies of structure-activity relationships (SARs) *in vitro *and preliminary evaluation *in vivo *[1].

Cyanoguanidines hyperpolarizing adenosine triphosphate (ATP)-sensitive potassium channels in the smooth muscle of the bladder have received attention as drug candidates for overactive bladder. One example is shown in Figure 1[2]. In addition to their appearance in pinacidil and other potassium channel openers (on which CHS 828 has no activity), substituted cyanoguanidines are also found in histamine-II receptor antagonists (e.g. cimetidine, N-cyano-N'-methyl-N''-[2-[[(5-methylimidazol-4-yl]methyl]thio]ethyl)guanidine) (Fig. 1).

As CHS 828 displays a broad spectrum of activity, low cross-resistance with standard drugs, favourable *in vitro *therapeutic indices [1,3,4] and IC_50 _values in the nano-molar range, the drug was brought into phase I clinical trials [5,6]. A detailed description of the mechanism of action of CHS 828 was not presented until 2004 when Dr Stangelshøj Olsen and co-workers published data supporting inhibition of the Inhibitory κ-B kinase (IKK) complex, with an IC_50 _of 8 nM [7]. The mechanism of action may, however, be dual, and may in part depend on drug concentration and exposure time as suggested by Hassan *et al *[8]. According to Ekelund *et al *[9], high concentrations (1 and 10 μM, well exceeding the IC_50_) of CHS 828, just like meta-iodobenzyl guanidine (MIBG), inhibit mitochondrial respiration, with a subsequent increase in glycolysis to cover the energy need. Interestingly, this high concentration effect is similar in resistant U-937/CHS cells and the U-937 GTB cells [9]. In addition, it was shown that cells grown in pyruvate-supplemented, glucose-free medium are, at least partly, protected from CHS 828 toxicity, thereby indicating that the extent of glycolysis may be an important determinant of optimal CHS 828 activity [9]. The process following CHS 828 exposure is characterized by an almost normal proliferation during the first 24 hours followed by an abrupt shut-off of deoxyribonucleic acid (DNA) and protein synthesis, a modest increase in caspase-3 activity and DNA fragmentation, and the first signs of cell death [10,11]. These processes seem dependent on intact protein synthesis [10]. Addition of the adenosine diphosphate (ADP) ribosylation inhibitor 3-aminobenzamide to the medium and U-937 cells exposed to CHS 828 resulted in a 100-fold decrease in IC_50_, and also in more predominant apoptotic cell death [12]. This effect, however, appears to be unrelated to the effects on poly(ADP-ribose) polymerase (PARP), and the authors suggest that it is mediated through effects on glycolytic enzymes, such as glyceraldehyde-3-phosphate [12]. In the subsequent paper it was shown that PARP-1 inactivation in mouse fibroblasts in fact *sensitizes *cells to the cytotoxic action of CHS 828, and consequently, that the drug is able to activate different cellular pathways depending on PARP status [13]. During the 2007 American Association for Cancer Research/National Cancer Institute/European Organization for Research and Treatment of Cancer (AACR-NCI-EORTC) International Conference on Molecular Targets and Cancer Therapeutics, Roulston and co-workers at Gemin X Biotechnologies Inc. presented data strongly suggesting that GMX1778 (EB1627), a soluble prodrug of CHS 828, acts by inhibition of nicotinamide phosphoribosyl transferase (Nampt), an enzyme involved in nicotinamide adenine dinucleotide (oxidized) (NAD+) biosynthesis, and that nuclear factor-kappa B (NF-κB) inhibition is only a consequence of NAD+ decline [14]. This mechanism was recently confirmed in a study by Dr Høgh Olesen and co-workers, including cross-resistance to FK866, a known inhibitor of Nampt [15]. Nampt has also been presumed to be a cytokine (PBEF) or a hormone (visfatin). The crystal structure of Nampt in the presence and absence of nicotineamide mononucleotide shows that Nampt is a dimeric type II phosphoribosyltransferase and provides insights into the enzymatic mechanism [16]. Previously noted effects by CHS 828 on glycolysis and inhibition of the transcription factor NF-κB can likely be secondary to inhibition of Nampt.

The aim of this study was to establish the structural requirements for cyanoguanidine cytotoxicity. The cytotoxic effects of nineteen substituted pyridyl cyanoguanidines, with variations in pyridyl substitution, different alkyl chain linkers and end groups, were studied on a panel of ten cell lines.

## Methods

### Cell lines

Cytotoxicity was assayed in a panel of ten human tumour cell lines. This panel was previously established for use in the early investigation of new anti-cancer compounds, and the cell lines represent different histological origins and mechanisms of resistance [17]. The panel has successfully been used in *in vitro *evaluation of a wide range of different cytotoxic agents, both for clinical and for investigational purposes. A brief description of the cell lines is presented in Table 1. Cells were maintained as previously described [17].

**Table 1:** Cell line characteristics

**Parental cell line**	**Sub-line(s)**	**Origin**	**Selecting agent**	**Proposed resistance mechanism**
CCRF-CEM	CEM/VM-1	Leukaemia	Teniposide	Topo II-associated
NCI-H69	H69/AR	SCLC	Doxorubicin	MRP
RPMI8226/S	8226/Dox40	Myeloma	Doxorubicin	Pgp
	8226/LR5		Melphalan	GSH
U-937 GTB	U-937 Vcr	Lymphoma	Vincristin	Tubulin-associated
ACHN	-	Renal		Primary resistant

GSH = glutathione; Pgp = p-glycoprotein 170; SCLC = small-cell lung cancer; Topo II = topo-isomerase II MRP = Multidrug resistance associated protein

### Medium and reagents

Cell growth medium RPMI-1640 (Sigma Chemical Co., St. Louis, MO, USA) supplemented with 10% heat-inactivated foetal calf serum (FCS) (Sigma), 2 mM glutamine, 100 μg/mL streptomycin and 100 U/mL penicillin was used for all experiments. Fluorescein diacetate (FDA) (Sigma) was dissolved in dimethylsulphoxide (DMSO) (Sigma) to a concentration of 10 mg/mL and kept frozen as a stock solution in the dark.

### Test compounds

The test compounds, a set of 19 structure analogues to CHS 828 with the cyanoguanidine core preserved, was a kind gift from the Department of Chemical Research, Leo Pharma, Ballerup, Denmark. Compounds were synthesized using methods described elsewhere [1,9].

### Cytotoxicity assay

The FMCA has been previously described in detail [18-20]. The method is based on 72-hour drug exposure in 96-well microtitre plates, whereafter cells are washed and the fluorescence generated from hydrolysis of fluorescein diacetate is measured. Fluorescence is proportional to the number of living cells in the well. The method has been used extensively for experimental as well as clinical purposes, and seems valuable with most classes of chemotherapeutic drugs. For concentration-response curves, each drug was tested at five different concentrations, each concentration in triplicate. Each 96-well microtitre plate had six control (unexposed cells) and six blank (cell medium only) wells. Quality criteria for a successful assay included > 90% starting viability (judged by Trypan blue exclusion), a control signal more than ten times the blank, and, finally, a coefficient of variation in control and blank wells of < 30%.

### Statistical analysis

Direct comparisons of the activity for the substances were made by using paired *t*-tests in GraphPad Prism, version 4 (Graphpad Software Inc., La Jolla, CA, USA).

## Results

The compounds showed varying cytotoxic activity allowing the SAR analysis; however, the dose-response curves for some analogues were very shallow, in several cases ending in a plateau at a survival index of 20–80%, thereby preventing a correct determination of the IC_50 _value. This characteristic dose-response profile has been previously described for CHS 828 in the fluorometric microculture cytotoxicity assay (FMCA) [3]. Prolonged assay time (violation to standard FMCA protocol) to 144 hours produced better dose-response curves and allowed determination of the IC_50 _values, results were similar but slightly changed (not shown). To avoid interference the survival index at 10 μM (on the plateau) was used with FMCA assay time 72 hrs (standard protocol). In a phase I trial reported by Ravaud *et al *[5] CHS 828 was given orally as a singe dose every 3 weeks. Doses up to 500 mg was given, which resulted in plasma concentrations of 11 μM. The inherent order of sensitivity among the cell lines was similar to that presented previously [3], with the doxorubicin resistant small-cell lung cancer (SCLC) cell line H69AR being the most sensitive and the maternal myeloma cell line RPMI 8226S being highly sensitive, while the doxorubicin-selected (p-glycoprotein (Pgp)-expressing) sub-line, 8226 Dox40, displayed a considerable degree of resistance, as did the T-cell leukaemia lines CCRF-CEM and CEM/R. This pattern of activity among the cell lines was similar for most of the compounds (see below). The reference compounds pinacidil and cimetidine (Fig. 1) were tested in concentrations of up to 10 mM in all ten cell lines and were found to be non-toxic (not shown).

**Figure 2:**
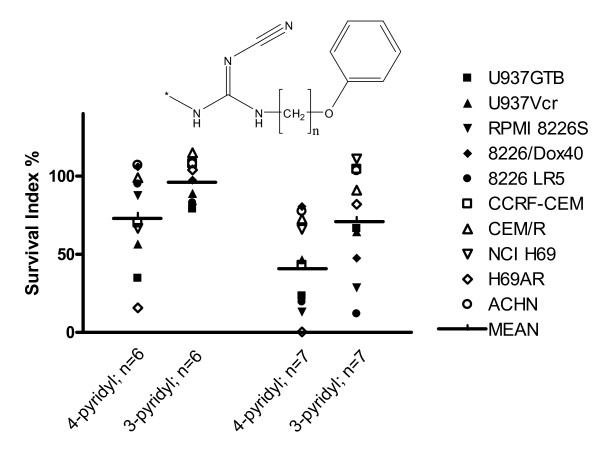
**Effects of pyridyl substitution pattern on cytotoxic activity (as survival index at 10 μM) in the ten cell lines studied**. The symbols for each cell line is used in figure 2–4.

First, the pyridyl substitution pattern on the cyanoguanidine core was studied. As seen in Figure 2, 4-pyridyl substitution was significantly more efficient than was 3-pyridyl substitution (p < 0.01).

**Figure 3:**
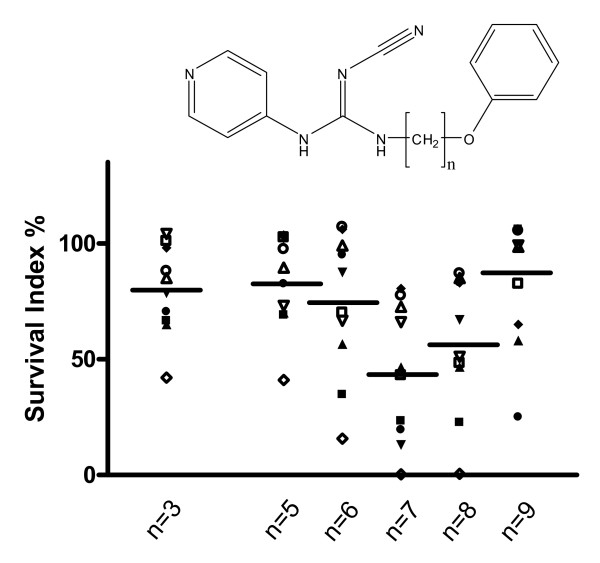
**Effects of linking chain length on cytotoxic activity (as survival index at 10 μM) in the cell lines studied**. Symbols as in figure 2.

**Figure 4:**
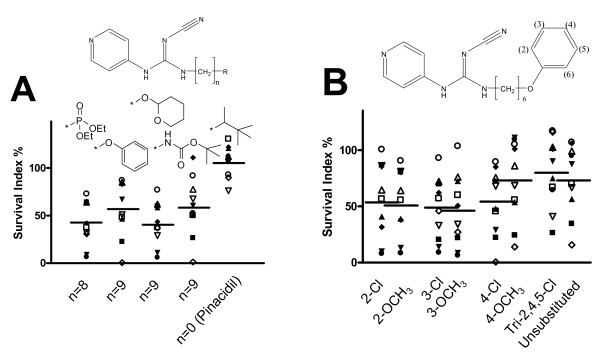
**Effects of different end groups (4A); and end group substituents (4B), on cytotoxic activity (as survival index at 10 μM) in the cell lines studied**. Symbols as in figure 2.

Second, the linking alkyl chain length was examined. This factor was apparently of considerable importance, having optimum activity with hexyl, heptyl or octyl chains ending with a bulky end group (Fig. 3). The exact composition of this end group did not seem to be of crucial importance (Fig. 4A). When the end group was a mono-substituted phenyl ring it was shown that the preferred position was 3-substitution, followed by 2- and, finally, 4-substitution, but differences were minor. The compound with an unsubstituted phenyl was as active as the 4-substituted compounds, while the trichlorinated derivative appeared less active (however, the difference was statistically not significant; p = 0.13). Whether the substituent(s) was a chloro- or methoxy-group(s) seemed to be of minor importance (Fig. 4B).

Finally, the activity patterns in the ten cell lines were compared. Substances with similar structures correlated well, whilst substances with large differences in molecular structure demonstrated lower correlation coefficients (not shown).

## Discussion

The unsubstituted pyridyl moiety appears important, although it has not been studied in detail in the present study. Others have shown that a substituted pyridyl or a phenyl substituent strongly reduces the cytotoxic activity of the compounds. Interestingly, however, *N*-(6-(4-chlorophenoxy)hexyl)-*N'*-cyano-*N''*-((2-methoxy)-5-pyridyl guanidine is a fairly potent inhibitor of the IKK activity *in vitro *(IC_50 _22 nM v. 8.0 nM for CHS 828), which contrasts with its low anti-proliferative activity against NYH SCLC cells *in vitro *and *in vivo *[7], and consequently in this particular case the cytotoxic action and the IKK inhibitory action appear separate. Furthermore, while retaining the capacity of increasing the glycolytic activity in U-937 cells, this compound appears to be almost atoxic also in this cell line (compound 8 in Ekelund *et al *[9]), again leading to speculations about a dual mechanism of action. Compounds with the pyridyl ring substituted for a mono or unsubstituted phenyl ring display no IKK inhibitory or cytotoxic activity [7]. Arylcyanoguanidines with short aliphatic side chains (e.g. *N*-aryl-*N'*-cyano-*N''*-alkyl-guanidines) are known as activators of ATP-sensitive potassium channels and inhibitors of insulin release from beta cells, but small structural changes can dramatically change the efficacy [21].

The length of the alkyl chain linking the cyanoguanidine to a bulky end group proved to be important, ideally being an hexyl to an octyl chain, in agreement with a previous report from Schou and co-workers [1]. In light of this, it is worth noting that the dodecyl derivative with a *tert*-butyloxycarbonylamino end group (CHS 850) nevertheless has shown high potency as an inhibitor of IKK activity, as well as proliferation of NYH SCLC cells *in vitro *and *in vivo *[7].

It has recently been shown that different Na^+^/H^+ ^exchange inhibitors, including the cyanoguanidine cimetidine, also inhibit chemokine production and NF-κB activation in immuno-stimulated endothelial cells [22]. For the model substance in these studies, amiloride (with no structural relationship to the cyanoguanidines), this was suggested to result from an inhibitory effect on IκB degradation [22]. Cimetidine and amiloride are both completely non-toxic in the FMCA model in the cell lines studied (Ekelund *et al *[23] and unpublished data), and interaction analysis between amiloride and CHS 828 in U-937 cells has shown synergistic effects [23].

In order to develop a second-generation drug candidate from CHS 828 with the aim to improve cytotoxicity and/or selectivity, Chern and co-workers synthesized analogues with a bridge connecting the two amino nitrogens of the cyanoguanidine, thus forming a cyclic structure such as 2-cyanoimino-4-imidazodinone derivatives. Some of these drugs, especially the 5-substituted ones, displayed cytotoxic activities at sub-micromolar level. However, in contrast to the analogue series presented in this paper, the rigid analogues appeared to display a different activity spectrum compared with the model compound (namely, higher activity against colon and liver cancer cell lines, and lower activity against gastric, nasopharyngeal and breast cancer) [24]. However, it is at present unclear whether these compounds with cyclic structures displays a more favourable activity profile than the compounds described in our work.

## Conclusion

The cytotoxic cyanoguanidines, with CHS 828 as the model compound, have received a fair amount of attention in the scientific literature, and early clinical trials have been performed. Further development resulted in a water soluble prodrug called EB1627 (also denoted GMX1777). Prolonged infusions of EB1627 have recently been reported to yield significant effects in myeloma, small cell lung cancer and colon cancer xenografts, which according to the authors support the design of an open-label, dose-escalation trial, in which patients with refractory solid tumors and lymphomas receive 24 h infusions of EB1627 as a single agent in 3-week cycles. Furthermore, results indicate that nicotinic acid is a potent antidote to treat EB1627 overdose [25].

Despite efforts for 10 years to pinpoint details of the mechanistic events following exposure to the drug, the exact mechanism of action was not established until very recently. Malignant cells display increased demands for energy production and DNA repair, and NAD is required for both processes and is also continuously degraded by cellular enzymes. *N*-(6-(4-chlorophenoxy)hexyl)-*N'*-cyano-*N''*-4-pyridyl guanidine most probably acts by inhibition of nicotinamide phosphoribosyltransferase (Nampt), a crucial factor in the resynthesis of NAD [14,15]. Using a functional non-clonogenic viability assay we identified structural prerequisites for cytotoxicity, and found CHS 828 to be a reasonable drug candidate.

## Authors' contributions

HL and JG contributed equally to the planning, the performance of the experiments and the analysis. RB was engaged in data analysis and methodology questions, including stability issues and additional laboratory work, upon major revision of the manuscript. The manuscript was mainly written by JG and reviewed by HL and RB.

## Declaration of competing interests

The authors declare that they have no competing interests.
